# Left Ventricular Noncompaction in Concomitance With Heroin Use Disorder

**DOI:** 10.7759/cureus.45366

**Published:** 2023-09-16

**Authors:** Yashitha Chirumamilla, Nihara Chakrala, Huda Marcus

**Affiliations:** 1 Internal Medicine, Hurley Medical Center, Flint, USA; 2 Internal Medicine, Hurley Medical Center - Michigan State University, Flint, USA

**Keywords:** left ventricular noncompaction cardiomyopathy, left ventricular noncompaction, heroin use, heroin use disorder, congenital cardiomyopathy

## Abstract

Left ventricular noncompaction (LVNC) is a rare congenital condition defined by the presence of prominent trabeculations in the myocardial layer of the left ventricle. The clinical presentation is varied as some patients are asymptomatic and others have symptoms of decompensated heart failure, arrhythmias, or thromboembolism. We present the case of a 42-year-old female with a past medical history of asthma and substance use disorder who presented to the Emergency Department following a syncopal event. The patient had used heroin intranasally, following which she became unresponsive for several minutes. Her husband witnessed the event and initiated chest compressions. When examined by emergency medical services (EMS), she had a palpable pulse and was given naloxone. The patient underwent further evaluation and was admitted for the treatment of aspiration pneumonia. Throughout her hospital stay, she complained of chest pain with musculoskeletal characteristics, likely secondary to chest compressions. However, due to the persistence of pain, she had further cardiac evaluation done. Her electrocardiography (EKG) revealed a normal sinus rhythm with no acute ischemic changes. Her echocardiography revealed left ventricular apical trabeculations with normal systolic and diastolic function, in line with the diagnosis of LVNC. Upon discharge, she was extensively counseled to abstain from substance use and to follow up with cardiology for a cardiac event monitor. Given her initial syncopal event and high-risk substance use behavior, she would benefit from close follow-up for the presence of arrhythmias.

## Introduction

Left ventricular noncompaction (LVNC) is a rare congenital cardiomyopathy characterized by a thin epicardial layer and a thicker endocardial layer with prominent trabeculations and deep recesses that communicate with the left ventricular cavity. The embryological origin of LVNC is thought to be due to failed compaction of the myocardial primordium. LVNC can exist without compromising the left ventricular function and has been identified in asymptomatic individuals incidentally. However, LVNC can also lead to dilated, hypertrophic, or restrictive cardiomyopathy. The precise prevalence of LVNC is not known but is estimated to be less than 1.4% of total individuals undergoing echocardiography. The prevalence is noted to be higher in individuals with a family history of the condition [1].

## Case presentation

**Figure 1:**
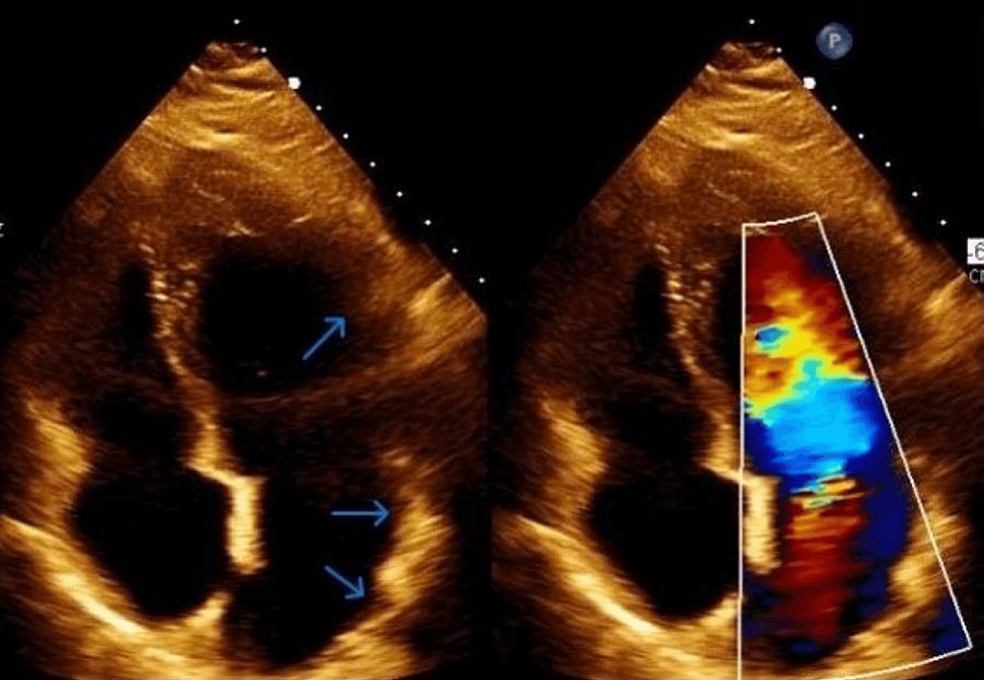
Echocardiography demonstrating multiple trabeculations (blue arrows) within the left ventricular cavity

We present the case of a 42-year-old Caucasian female with a past medical history significant for asthma and substance use disorder presented to the Emergency Department following an episode of loss of consciousness. The patient’s husband reported that she had used heroin intranasally, following which she became unresponsive. Her husband initiated chest compressions for several minutes while awaiting the arrival of the ambulance. During her first evaluation by emergency medical services (EMS), she did have a palpable pulse. En route to the hospital, she was placed on a nonrebreather mask due to respiratory distress and was given 8 mg of naloxone. Upon arrival at the Emergency Department, the patient was alert and oriented to time, place, and person. Her blood pressure and heart rate were within normal limits, and she was afebrile. Her oxygen saturation improved, and she transitioned to oxygen via nasal cannula. Chest radiography revealed mildly prominent perihilar markings. Computed tomography of the chest showed bilateral lower lobe consolidations, suggestive of pneumonia. The patient was admitted to the General Medicine floor for further treatment of aspiration pneumonia. During her hospital stay, she continued to complain of reproducible chest pain localized to the mid-sternum. She denied accompanying symptoms such as shortness of breath and nausea and did not have characteristics typical of cardiac chest pain such as worsening with exertion. Although her pain was believed to be musculoskeletal in nature secondary to chest compressions, due to the persistence of the chest pain she had further workup. An electrocardiogram (EKG) revealed normal sinus rhythm with no acute ischemic changes and troponin values were within normal limits. Echocardiography using contrast revealed left ventricular apical trabeculations with normal systolic and diastolic activity and an ejection fraction of 55-60% (Figure 1).

She was diagnosed with isolated LVNC. Upon discharge, she was thoroughly counseled regarding abstinence from illicit substances and advised to follow up with cardiology for cardiac event monitoring and close monitoring for potential arrhythmias or development of cardiomyopathy.

## Discussion

The classification of LVNC is still highly debated. The European Society of Cardiology describes it as a “non-classified cardiomyopathy,” while the American Heart Association uses the term “genetic cardiomyopathy.” Other subtypes have also been proposed, which include isolated LVNC, with noncompacted morphology and normal systolic and diastolic function and noncompaction cardiomyopathy, with left ventricular dilation and dysfunction. With no true consensus regarding the terminologies and “gold standard” diagnostic method, the true incidence of the condition is skewed as it is often overdiagnosed [2]. Diagnostic criteria for LVNC can be specific to the imaging modality being utilized. Jenni criteria and Chin criteria are commonly utilized with echocardiography. Petersen criteria can be used with cardiac magnetic resonance imaging (MRI). All of the criteria generally involve the presence of prominent trabeculations along with specified ratios of compacted and non-compacted myocardium [3]. Our patient's diagnosis would also be further confirmed with cardiac MRI as the diagnostic criteria are clearer.

Clinical presentation at the time of diagnosis is highly variable. The three most common presentations are with symptoms of heart failure, arrhythmias including atrial fibrillation, bundle branch blocks, ventricular tachycardias or sudden death, and thromboembolism. Coronary artery disease has also been described in various case reports as the initial manifestation [4]. Many patients are diagnosed incidentally and are largely asymptomatic as well. Our patient’s initial symptom was loss of consciousness, which was thought to be secondary to her heroin use; however, it could have been an arrhythmia leading to syncope. 

The follow-up for patients diagnosed with LVNC should include screening of the patient’s family members and possibly further genetic testing. Up to 12-50% of patients had immediate relatives affected by the condition with variable inheritance patterns including autosomal dominant, recessive, and X-linked [1]. There are currently no specific management guidelines for LVNC. The recommendation is to treat each individual presentation, such as heart failure or thromboembolism. Anticoagulation is not universally recommended for patients with LVNC but is recommended in those with reduced ejection fraction as they are at a higher risk for thrombus formation [5]. Routine implantable cardioverter-defibrillator (ICD) implantation is also not recommended and should only be performed if other indications exist. Given her presentation, our patient was advised to wear a cardiac event monitor for an extended period to evaluate for life-threatening arrhythmias. A recent meta-analysis concluded that patients with LVNC have the same risk for cardiovascular or all-cause mortality as those with dilated cardiomyopathy (DCM) and the most significant prognostic factor is ejection fraction, not the burden of trabeculations [6]. However, a major risk factor for cardiovascular events that should be considered in our patient’s case is heroin use. There is currently no literature describing the adverse effects of heroin or other drug use in conjunction with LVNC. Although our patient is not at an increased risk given her normal ejection fraction, her heroin use disorder deems it necessary for her to have a consistent follow-up.

## Conclusions

Following the diagnosis of LVNC, adequate follow-up with cardiology is crucial, especially in the presence of other cardiovascular risk factors, such as substance use. The recommended approach in the management of LVNC is understanding the different presentations of the condition and treating each accordingly. As arrhythmias are a common presentation of LVNC, patients with complaints of syncopal events should be thoroughly monitored so that potentially life-threatening arrhythmias can be identified. Hypertrabeculation associated with LVNC also increases the risk of clot formation and patients could present with thromboembolism. Hence, anticoagulation is a recommendation in LVNC with reduced ejection fraction. The association between heroin use and increased cardiovascular risk is established, but substance use in conjunction with LVNC has not been previously studied. Thus, the increased risk of this particular association is not yet well established. Further studies regarding long-term outcomes of patients with substance use disorder and LVNC could help establish more specific guidelines in management.
